# Heterogeneity among traumatic spinal cord injuries at the thoracolumbar junction: helping select patients for clinical trials

**DOI:** 10.1038/s41393-019-0317-x

**Published:** 2019-06-25

**Authors:** Shu-Jia Liu, Qiang Wang, He-Hu Tang, Jin-Zhu Bai, Fang-Yong Wang, Zhen Lv, Shi-Zheng Chen, Jie-Sheng Liu, Yi Hong, Jun-Wei Zhang

**Affiliations:** 1 Faculty of Rehabilitation Medicine, Capital Medical University, Beijing, China; 2 Department of Spine and Spinal Cord Surgery, China Rehabilitation Research Center, Beijing, China; 3 Department of Anesthesiology, China Rehabilitation Research Center, Beijing, China

**Keywords:** Spinal cord diseases, Spinal cord diseases

## Abstract

**Study design:**

Retrospective analysis.

**Setting:**

China Rehabilitation Research Center, Beijing, China.

**Objective:**

A retrospective study that documents the modalities and clarifies the heterogeneity among spinal cord injuries (SCIs) caused by trauma to the thoracolumbar vertebral junction.

**Methods:**

X-ray and MRI imaging, neurological records, and the urodynamics results of 190 patients were reviewed and used to categorize different SCI modalities. First, injuries were divided into complete and incomplete injuries using the International Standard for Neurological Classification of Spinal Cord Injury. Next, the complete injuries were further grouped using the neurological level of injury and Long T2 signal from mid-sagittal MRI images, whereas the bulboconvernosus reflexes were also used as a reference to detect injury to the sacral cord.

**Results:**

The SCI modalities were classified into five categories: pure complete epiconus lesion with caudal cord intact (G1), complete epiconus injury with conus medullaris (CM) totally involved in the lesion (G2), CM syndrome, cauda equine syndrome without sacral sparing (G3 and G4), and incomplete injury (G5).

**Conclusions:**

The heterogeneity of SCIs at the thoracolumbar junction was documented, a criterion we propose to be of great significance when selecting patients for clinical trials. In particular, the G2 group, which comprises nearly one third of the patients with epiconus lesions, is sometimes mistaken as G1, an observation that has thus far received insufficient attention.

## Introduction

Most spinal trauma occurs at the thoracolumbar junction (T11–L2), where the distal spinal cord, conus medullaris (CM), and cauda equina (CE) are located [1–4]. The CM, an intumescence of the caudal spinal cord that is a transition between the central and the peripheral nervous system, is known to have a variable location between T12 and L2 vertebral levels [5]. When injured, the neurological deficits resulting from damage to the CM is varied. Despite the extensive documentation regarding the evaluation and management of thoracolumbar spinal fracture and dislocation [6–9], few studies have adequately characterized the concomitant spinal cord injury (SCI) modalities. On the other hand, the rapid development of neural regeneration techniques has many implications for the treatment of thoracolumbar SCIs. Laboratory studies have shown that paraplegic rats that were subjected to a full repair strategy had most of their hind limb motor functions return [10]. However, numerous clinical trials performed in recent decades have resulted in limited progress in the treatment of human SCI [11–17]. One reason for this may be the lack of accounting for the heterogeneity in human SCI patients, as this often leads to underpowered clinical studies, the reporting of unreliable therapeutic effects, or the dismissing of useful therapies [18]. Therefore, before new SCI treatments can be brought to clinical trials, it is necessary to distinguish and categorize the pathological aspects of traumatic SCI at the thoracolumbar junction.

## Methods

Two hundreds and fifty-four patients with traumatic SCI at thoracolumbar junction were consecutively admitted in our department between January 2011 and February 2017, of which 190 patients were included in the present study, whereas 64 were excluded because they could not afford or refused to undergo urodynamic tests. The study population consisted of 131 males and 59 females, with an age ranging from 16 to 78 years old (see the supplementary file). All patients underwent spinal stabilization surgery within 1 week after injury, some of which were operated in our department, whereas the rest in other hospitals. They all started rehabilitation training in 3 weeks. The length of stay ranged from 3 to 5 months. The inclusion criteria were as follows: all patients had undergone imaging (X-ray, MRI), and data were present; their medical records included the results of video-urodynamics testing; and patients had filled out neurological assessment sheets according to the International Standards for Neurological Classification of Spinal Cord Injury (ISNCSCI) [19, 20]. The assignment of patients to different injury modality groups was performed by a comprehensive review of all of the patients’ clinical materials obtained from admission through to discharge. The study was carried out as follows:

### Radiological work-up

For each case, the vertebral level of injury was confirmed using X-ray images. The mid-sagittal T2 weighted MRIs, taken within 1 week after injury, were then scrutinized. After the location of CM was identified, the anatomical location of the of cord lesion—located above, in, or below the CM, or a combination of all locations—could be identified by the presence and range of the high-intensity signal (HIS).

### Neurological assessment

The ISNCSCI sheets, as well as the medical records of each case, were carefully reviewed. Any notes on abnormal motor function, deep tendon reflexes, sacral sparing, preservation, or early returned bulbocavernosus reflex (BR) were carefully checked and documented. Upper or lower motor neuron injuries, or a combination of the two, were identified.

### Grouping of patients

**Fig. 1:**
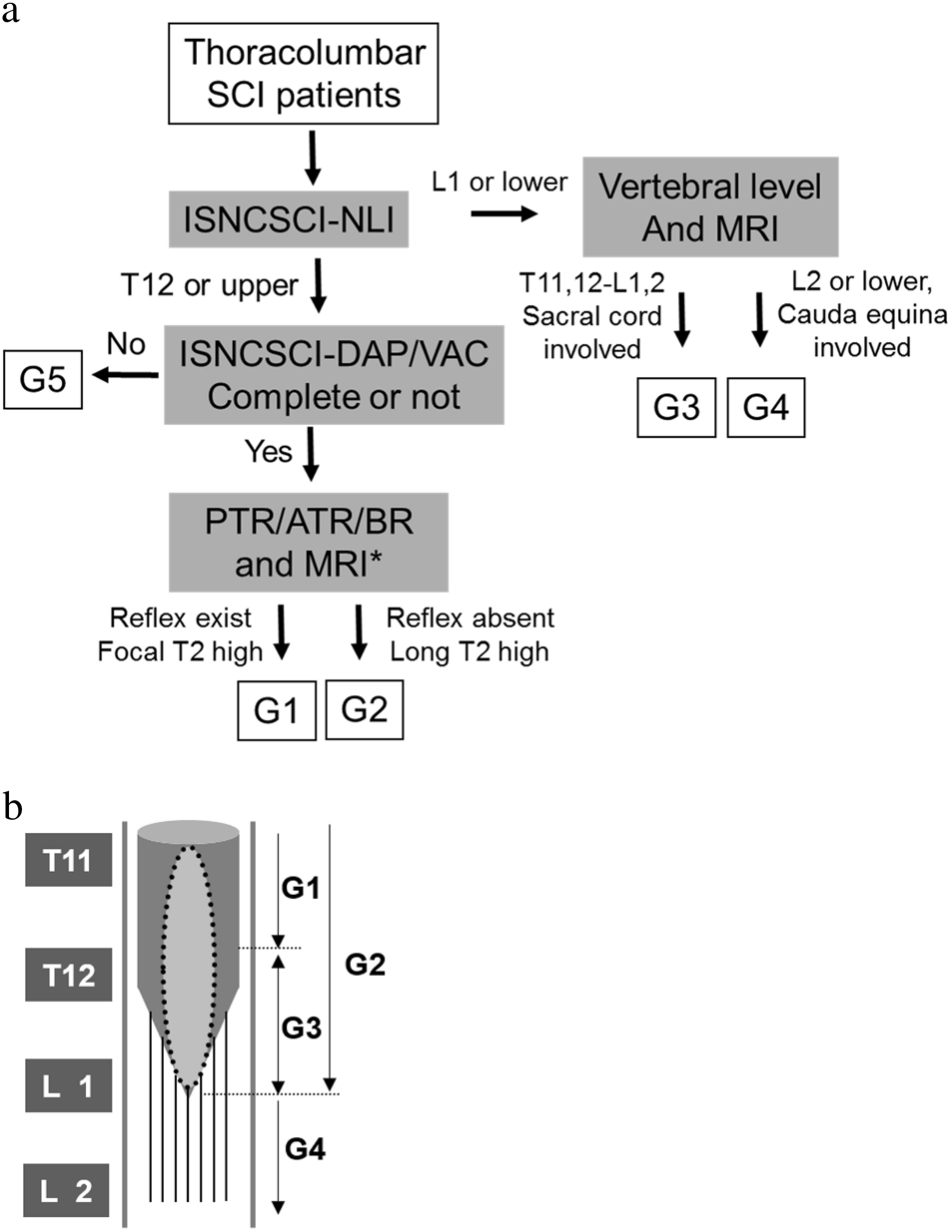
**a** Algorithm for grouping patients. ISNCSCI: International Standard for Neurological Classification of Spinal Cord Injury, NLI: neurological level of injury, DAP: deep anal pressure, VAC: voluntary anal contraction, PTR: patellar tendon reflex, ATR: Achilles tendon reflex, BR: bulbocavernosus reflex. *: not necessarily, just for reference. **b** The schema of the pathological modalities of SCI caused by thoracolumbar junction trauma. The dark gray cylinder with cone represents the spinal cord. The light gray spindle circled in a black dotted line refers to the long T2 signal, indicating the range of the cord lesion. G1: pure epiconus injury. G2: epiconus injury with caudal cord completely involved. G3: conus medullaris syndrome. G4: cauda equina syndrome. The gray blocks at the left side indicate the vertebral bodies of thoracic 11 to lumbar 2

The patients were then grouped into SCI modalities according to their above-mentioned evaluation. First, the SCI modalities were divided into complete and incomplete injuries using the relevant ISNCSCI guidelines. Next, cases of complete injury were further grouped by the neurological level of injury (NLI) and the existence and range of Long T2 signals in mid-sagittal MRI images, namely none, local, or extending to the tip of the cord. The deep tendon reflex and BR were also used to classify whether the cord below the NLI was totally involved in the injury (Fig. 1a).

### Analyzing the results of video-urodynamics

Video-urodynamics testing was performed in all patients at 1–3 months post injury using TRITON (Laborie, Canada), according to the manufacturer’s guidelines. The cystometry fill phase ceased when the case felt strong desire to void or urine leakage was found. The bladder capacity and maximal intravesical filling pressure (IVPmax) at the termination of the fill phase were obtained. The residual volume was determined by transurethral catheterization after bladder voiding had ceased. Cystourethrogram was done at several time points during filling and voiding phase according to the urologist’s instructions.

### Review of complications

Common complications such as pressure sores, deep vein thrombosis (DVT), spasticity, versicourethral reflux (VUR), heterotopic ossification (HO), lung infection, joint contracture, neurogenic pain, and amyotrophy were investigated in all patients from the onset of the injury until their discharge. The differences between the incidences of each complication among the groups were analyzed.

### Statistical analysis

Statistical Package for the Social Sciences (SPSS, version 19.0, SPSS Inc., Chicago, IL, USA) was used to analyze data. Continuous data are reported as the mean ± standard deviation. Chi-squared tests or a one-way analysis of variance were used to test for differences between categorical variables. A *P* value < 0.05 was considered statistically significant for all analyses.

## Results

### Modality categories

One hundred and ninety patients were assigned into five groups, according to their SCI modality, as analyzed by the processes described above (Table 1). The complete injury group included G1 (37 cases): pure complete epiconus injury with CM intact; G2 (43 cases): complete epiconus injury with lesion extending from the lower thoracic spinal cord to the tip of the CM; G3 (36 cases) and G4 (18 cases): CM syndrome (CMS, 36 cases) and CE syndrome (CES, 18 cases) as described in the 2011 ISNCSCI. The incomplete epiconus SCI (NLI: T9 to T12) group included G5 (56 cases): spinal cord below the NLI partially involved, and existence of the BR. In this group, a line of HIS could sometimes be found in the spinal cord, whereas mixed upper and lower motor neuron injuries could be seen. In addition, the loss of motor and sensory function, as well as bladder disorders, were varied and hard to characterize.

**Table 1:** Modalities of the 190 cases of traumatic spinal cord injury at the thoracolumbar junction

Group	*n* (%)	NLI	AIS	Neurological syndrome	HIS (MRI–T2)	Injury type	BR & AR
G1	37 (19.5)	T9–T12	A	Epiconus	Focal	UMN	(+)
G2	43 (22.6)	T9–T12	A	Epiconus+CM	Extensive	LMN	(−)
G3	36 (18.9)	L1 to S4–5	A	CM	Focal	LMN	(−)
G4	18 (9.5)	L2 to S4–5	A	CE	None	LMN	(±)
G5	56 (29.5)	T9 to T12	B–D	atypical	Uncertain	mixed	(±)

*NLI* neurological level of injury, *AIS* ASIA impairment of scale, *HIS* high-intensity signal, *BR & AR* bulbocavernosus reflex and anal reflex, *CM* conus medullaris, *CE* cauda equine, *UMN* upper motor neuron, *LMN* lower motor neuron

**Fig. 2:**
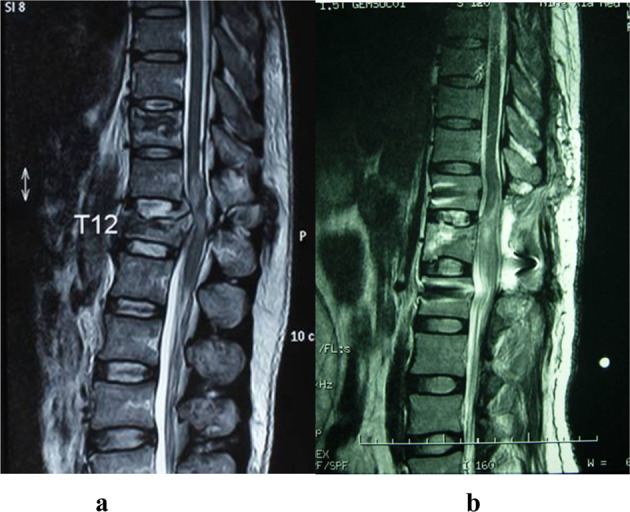
Typical mid-sagittal views of MRI images of the patients in G1 and G2, showing the focal or extensive long T2 signals in the spinal cord. **a** Male 25 yrs, from G1, focal long T2 signal was found only at the spine trauma level, showed limited the cord lesion. **b** Female, 40 years-old, from G2 L1 burst fracture, a typical T10 SCI, with long T2 signals found at the injury level extended to the tip of the cord, indicating the wide range of cord injury

The morphological aspects of the pathoanatomical ranges of the G1, G2, G3, and G4 groups is shown in Fig. 1b. HIS seen in the MRIs of typical G1 and G2 patients are shown in Fig. 2a, b.

### Results of video-urodynamics tests

**Fig. 3:**
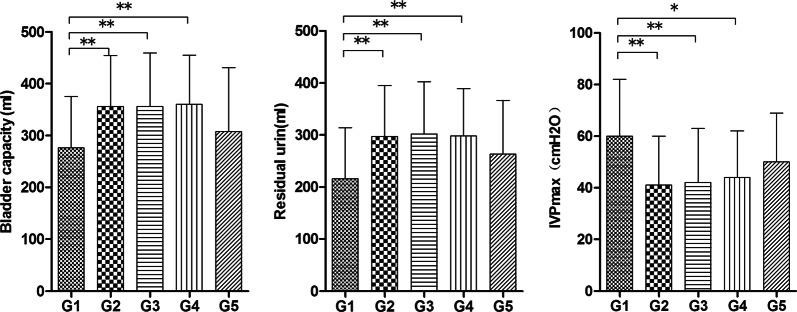
Comparison of video-urodynamics parameters among the five groups. The values of the three measurements of the five groups were compared. All data are expressed as means ± SD. *p < 0.05, **p < 0.01, according to one-way ANOVA

The bladder capacity and residual urine volumes of G1 patients (276 ± 99 ml and 216 ± 98 ml) were significantly lower than those of G2, G3, and G4 (*p* < 0.01), yet the inverse was true for the IVPmax (*p* < 0.05, 0.000–12). The residual urine volumes and IVPmax of all groups were higher than 200 ml and 40 cm H_2_O, respectively. The values of the three measurements of the G5 group were not significantly different than those of the other four groups (*p* > 0.05) (Fig. 3). Seven patients from G1 and one or two patients from every other group were reported to have a VUR.

### Complications

Different kinds of complications occurred in G1 and G2 group patients. Incidences of DVT in the lower extremities and amyotrophy were significantly higher in the G2 group than in the G1 group, whereas VUR, spasticity and joint contracture occurred more frequently in G1 patients (*P* < 0.05; Table 2).

**Table 2:** Incidence of complications in the G1 and G2 groups

Complications	G1 (*n* = 37)		G2 (*n* = 43)		*X* ^2^	*P*
	*n*	%	*n*	%		
Pressure sore	1	3.125	2	5.263	0.000 (correction)	1.000 (correction)
DVT	1	3.125	9	23.684	4.435 (correction)	0.035 (correction)
VUR	7	21.875	1	2.632	4.596 (correction)	0.032 (correction)
Lung infection	2	6.250	2	5.263	0.000 (correction)	1.000 (correction)
Joint contracture	5	15.625	0	0.000	4.255 (correction)	0.039 (correction)
Spasticity	19	59.375	0	0.000	30.968	2.623 × 10^−8^
Neurogenic pain	5	15.625	5	13.158	0.000 (correction)	1.000 (correction)
amyotrophy	4	12.500	35	92.105	44.616	2.397 × 10^−11^
HO	2	6.250	3	7.895	0.000 (correction)	1.000 (correction)

*DVT* deep vein thrombosis, *VUR* versicourethral reflux, *HO* heterotopic ossification

## Discussion

The nature of the neurological deficits after a traumatic injury to the spinal cord can be predicted from the lesion site at cervical, or upper and middle thoracic levels. In clinical practice, however, at the thoracolumbar junction the lower thoracic and lumbar cord, the CM, and the lumbar and sacral roots are all located within a limited area. As a result, destructive forces can lead to extended tissue damage, complicating the neurological patterns of the lesion [10, 18, 21, 22]. On the other hand, the rapid development of neural regeneration techniques has many implications for the treatment of thoracolumbar spinal injuries. Unfortunately, although numerous clinical trials have been performed in recent decades, they have resulted in limited progress in the treatment of SCI [18]. A lack of acknowledgment of the heterogeneity in thoracolumbar injuries may lead to underpowered clinical studies. As a result, studies may misstate the existence of a therapeutic effect or dismiss useful therapies, denying patients access to potentially beneficial therapies. Therefore, prior to clinical trials, it is necessary to classify the pathoanatomical and pathophysiological aspects of the neural lesions.

In this study, thoracolumbar junction SCIs were first roughly classified into three categories according to the NLI; namely the epiconus (T9–T12), CMS and CES, as has been performed in previous studies [10]. However, the patients in the epiconus injury group, although given the same NLI, appeared to have many disparities in their clinical manifestations, such as voiding dysfunction and motor/sensory disturbances. In the present study, considerable effort was thus made to investigate the heterogeneity within the epiconus injury group. First, the “pure” complete epiconus injury patients were separated and grouped as G1. Next, the cases of complete injury, with lesions extending from the lower thoracic spinal cord to the tip of the conus, were classified as G2. The distinction between G2 and G3 is not an anatomic/morphologic one but rather one based on neurologic level of injury. If the NLI is T12 or above, we considered this to be a G2 injury, regardless of the MRI appearance. If the NLI is L1 or below, we considered this to be a G3 injury. Third, patients with incomplete epiconus SCI whose clinical manifestation was variable and with generally unpredictable outcomes, were assigned as G5. Most patients in G5 were a grade C on the ASIA Impairment Scale (AIS). Thus, it was not enough to distinguish one from another using the NLI and AIS, as the degree of motor function preserved and disorders of urination and defecation were quite varied. Using these cases for clinical trial, the homogeneity of cases is hard to obtain, whereas the prognosis will be influenced by additional factors such as differing degrees of spontaneous recovery.

Voiding dysfunctions after trauma to the thoracolumbar junction have previously been characterized by Chuang et al. [10], where they assigned patients into three groups according to injury patterns: epiconus, CM, and CE injury. However, the characteristics of the groups were not clearly distinguished, which may be attributed to the fact that patients, such as those in G2 and G5 in our study, were not separated from the epiconus lesion group. In Chang et al.’s study, the bladder structure, including smooth muscle and connective tissue, in the CE injury group were found to be still functioning. This was also found in the present study in groups with injury to the CM and CE. Surprisingly, we also found that bladder capacity and residual urine volumes reduced faster than expected in the G1 group. As our patients were evaluated at a relatively acute phase of SCI, these observations suggest that solutions to prevent bladder contracture should be implemented as early as possible. The mean IVPmax in all groups was above 40 cm H_2_O, which may increase the risk of renal damage. Thus, if clean intermittent catheterization is implemented, it should be done at an appropriate time interval.

It is necessary to classify thoracolumbar SCIs not only according to ISNCSCI guidelines, but also by the pathoanatomical structure of the injury. This will aid in patient management decisions and prognostication, and will also enhance a study’s chance of identifying the true effect of treatment, while minimizing the risk of misattributed treatment effects. Most clinical trials have failed to demonstrate effective therapies for acute traumatic SCI; this translational failure may be attributed to insufficient attention paid to the heterogeneity of SCIs, and an under-appreciation of the impact of the most important baseline prognostic variables [18]. Dvorak et al. stratified a clinical trial cohort using the joint variables of the NLI and the AIS, and patients were further divided into subgroups of high cervical, low cervical, thoracic (T2–T10), or thoracolumbar (T11–L2) injuries [18, 23]. A significant difference in motor recovery between thoracic and thoracolumbar injury groups was found when the initial AIS grade was A. This is unsurprising, given the unique neuroanatomy of the region, and given that the degree of neurological recovery has been shown to be dependent upon regional differences in spinal cord anatomy. Kingwell et al. [24] reported the heterogeneity of thoracolumbar SCI in 2011. They introduced a method of determining the termination of the CM and the precise neural axis level of injury by utilizing MRI, and concluded that the motor recovery of patients with a thoracolumbar spinal injury and a neurological deficit was affected by both the neural axis level of injury as well as the initial motor score. According to our experience, however, as far as the impact of the recovery of motor function, the influence of axial range of cord injury, usually appeared clinically as AIS, was relatively clear, but the longitudinal range of neural axis injury was uncertain. As this study focus on the latter, the observation of motor function recovery was not included. The heterogeneity of the thoracolumbar group in Dvorak et al.’s study was identical to what we found in the epiconus injury group in the present study. This indicates that patients in the G1 and G2 groups should be separated in subsequent studies. Based on the above discussion, each group of patients in this study could be candidates for a clinical trial. We emphasize here that they should be recruited separately, not mixed.

Common complications of SCI were seen in our patient cohort, similar to those reported in other studies. We focused on the different kinds of complications occurring in patients in the G1 and G2 groups. Given the same NLIs, patients in the G2 group suffered both upper motor neuron (UMN) and lower motor neuron lesions, and were likely to have DVT in the lower extremities, as well as amyotrophy. On the contrary, among patients in the G1 group, pure UMN injuries were found, and a high incidence of VUR, spasticity and joint contracture was seen. Thus, early intervention to prevent bladder contracture and appropriate management for muscle tone should be adopted.

## Conclusion

In this study, heterogeneity in the neurological lesions caused by thoracolumbar junction trauma was assessed and divided into five categories. The G1 group, consisting of pure epiconus injury, is most suitable for clinical studies. The G2 group, occupying nearly one-third of the epiconus injuries, was considerably different from the G1 group in terms of in neurological deficits, voiding dysfunction, and susceptibility to complications. This type of SCI has been commonly reported, but has received insufficient attention and characterization.

## Supplementary information

supplemental material

## Data Availability

See the supplementary file.
